# Mapping the Landscape of Mesenchymal Stem Cell-Derived Extracellular Vesicles: From Bench to Bedside

**DOI:** 10.1155/sci/5361754

**Published:** 2025-10-13

**Authors:** Nadiar M. Mussin, Kulyash R. Zhilisbayeva, Akmaral Baspakova, Madina A. Kurmanalina, Amin Tamadon

**Affiliations:** ^1^Department of General Surgery No. 2, West Kazakhstan Marat Ospanov Medical University, Aktobe, Kazakhstan; ^2^Department of Languages, West Kazakhstan Marat Ospanov Medical University, Aktobe, Kazakhstan; ^3^Department of Epidemiology, West Kazakhstan Marat Ospanov Medical University, Aktobe, Kazakhstan; ^4^Department of Dental Disciplines and Maxillofacial Surgery, West Kazakhstan Marat Ospanov Medical University, Aktobe, Kazakhstan; ^5^Department of Natural Sciences, West Kazakhstan Marat Ospanov Medical University, Aktobe, Kazakhstan

**Keywords:** exosomes, extracellular vesicles, immunomodulation, mesenchymal stem cells, regenerative medicine

## Abstract

Mesenchymal stem cell (MSC)-derived extracellular vesicles (EVs), including exosomes, have garnered significant attention for their therapeutic potential in regenerative medicine and inflammatory disease management. This bibliometric analysis maps the global research landscape of MSC-derived EV studies from 2014 to 2025, utilizing data from Web of Science (WoS), Scopus, and PubMed. A total of 99 research articles were analyzed after screening 335 initial records, focusing on publication trends, citation metrics, collaboration networks, and thematic evolution. The field exhibited a 27.11% annual publication growth rate, with 841 authors contributing to 70 journals, reflecting its interdisciplinary and collaborative nature. Key findings include a surge in publications from 2018 onward, driven by clinical trials targeting conditions such as COVID-19, osteoarthritis, and spinal cord injury. The United States and China led international collaborations, with 22.68% of publications involving cross-border co-authorships. Keyword analysis revealed a shift from foundational terms like “exosomes” to clinical applications like “immunomodulation” and “osteoarthritis.” Despite the field's promise, limitations such as partial 2025 data and exclusion of non-English studies suggest areas for broader inclusion. This study underscores the rapid growth and clinical potential of MSC-derived EV research, providing insights for researchers and policymakers to advance therapeutic development.

## 1. Introduction

Extracellular vesicles (EVs), particularly exosomes, derived from mesenchymal stem cells (MSCs) have garnered significant attention as a novel therapeutic platform due to their ability to mediate intercellular communication, modulate immune responses, and promote tissue repair [1]. MSCs, sourced from bone marrow, adipose tissue, umbilical cord, and other tissues, release EVs containing bioactive molecules such as microRNAs, proteins, and lipids, which exert therapeutic effects in various diseases, including cancer, neurological disorders, and inflammatory conditions [2]. Unlike cell-based therapies, EVs offer advantages such as lower immunogenicity, ease of storage, and potential for targeted delivery [3].

The field of MSC-derived EV research has expanded rapidly, with studies spanning in vitro models, in vivo animal models, and human clinical trials. In vitro studies have elucidated mechanisms of EV action, while in vivo studies have demonstrated efficacy in disease models such as myocardial infarction, osteoarthritis, and acute kidney injury [4]. Human clinical trials, though limited, have explored EV safety and efficacy in conditions like COVID-19, Crohn's disease, and Alzheimer's disease [5]. Despite these advancements, a comprehensive understanding of the research landscape, including key contributors, thematic trends, and translational gaps, remains limited.

Bibliometric analysis provides a quantitative approach to evaluate research trends, collaboration patterns, and impact within a field. This study conducts a bibliometric analysis of MSC-derived EV research, focusing on in vivo, in vitro, and human studies, to map its intellectual structure, identify research hotspots, and assess its translational potential. The findings aim to inform researchers, clinicians, and policymakers on advancing EV-based therapies.

## 2. Methods

### 2.1. Data Collection and Processing

A bibliometric analysis was performed using Web of Science (WoS), Scopus, and PubMed databases, with searches conducted in March 2025. The search strategy (Table 1) targeted MSC-derived EV research in in vivo, in vitro, and human studies, using Boolean and wildcard operators to capture relevant terms. The initial search retrieved 335 documents (WoS: 73; Scopus: 235; PubMed: 28).

**Figure 1:**
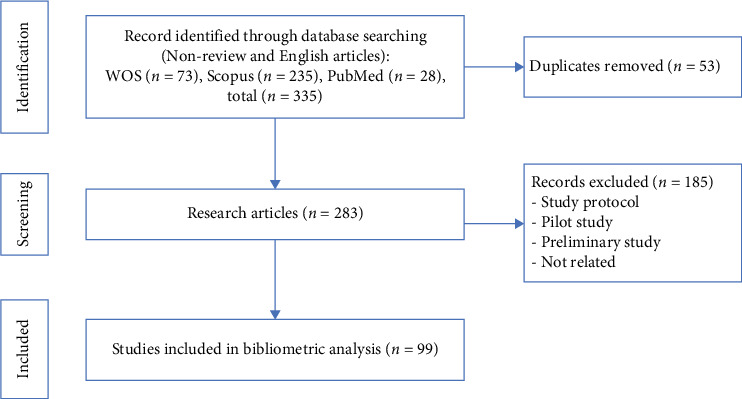
Flowchart of literature search and screening process for bibliometric analysis of mesenchymal stem cell-derived extracellular vesicles research.

Filters were applied to retain English-language research articles, excluding reviews, study protocols, pilot studies, preliminary studies, and nonrelevant publications. After screening, 283 research articles were identified, and 53 duplicates were removed. The final dataset comprised 99 articles, which were screened for relevance based on titles and abstracts. Metadata (titles, authors, affiliations, keywords, citations, and publication years) were exported in BibTeX and text formats and processed in RStudio (version 2024.12.1) using the bibliometrix package. The literature search and screening process is depicted in Figure 1.

### 2.2. Bibliometric Analysis Methods

The bibliometrix R package (version 4.5.0) and Biblioshiny were used to analyze publication trends, citation metrics, co-authorship networks, and keyword co-occurrence. Performance metrics included annual publication growth, leading authors, institutions, and journals. Science mapping visualized collaboration networks, keyword clusters, and thematic evolution. RAWGraphs was used for enhanced network visualizations. No ethical approval was required as the study used publicly available data.

## 3. Results

### 3.1. Characteristics of Publication Outputs

The bibliometric analysis encompassed 99 documents published between 2014 and 2025, sourced from 70 distinct journals, reflecting the multidisciplinary nature of MSC-derived EV research. The dataset primarily consisted of research articles, with no single-authored documents, indicating a highly collaborative research environment. The annual growth rate of publications was 27.11%, demonstrating a rapid expansion in the field over the 11-year period, likely driven by increasing interest in EV-based therapeutics. The average document age was 2.78 years, suggesting that the majority of publications are recent, aligning with the field's emerging status. Each document received an average of 52.76 citations, indicating significant scholarly impact and visibility within the scientific community.

A total of 841 authors contributed to the dataset, with an average of 9.92 co-authors per document, underscoring the collaborative nature of MSC-derived EV research. Notably, 22.68% of publications involved international co-authorships, highlighting robust global collaboration networks, particularly among researchers in North America, Asia, and Europe. The absence of single-authored documents further emphasizes the reliance on multiauthor teams, likely due to the complexity of EV research, which integrates expertise from stem cell biology, molecular biology, and clinical translation.

The dataset included 285 author-provided keywords and 1905 Keywords Plus (automatically generated by databases), providing a rich source for thematic analysis. These keywords reflect the diverse focus areas within the field, ranging from EV biogenesis to therapeutic applications in cancer, regenerative medicine, and inflammatory diseases. The high number of Keywords Plus suggests a broad interdisciplinary scope, as databases assign terms to capture related concepts, enhancing discoverability across related fields such as nanotechnology and immunotherapy.

### 3.2. Annual Scientific Production and Citation Trends

The publication output in MSC-derived EV research exhibited significant growth from 2014 to 2025, reflecting the field's rapid development. The dataset began with a single article in 2014, increasing steadily to a peak of 20 articles in both 2023 and 2024, and slightly decreasing to 14 articles in 2025, likely due to the partial year data (upto March 2025). This trajectory corresponds to the annual growth rate of 27.11% reported in Section 3.1, underscoring the burgeoning interest in EV-based therapeutics.

Citation analysis revealed varied impact across the years. The year 2014, with only one publication, had the highest mean total citations per article (599.00), likely due to its early contribution to a nascent field, allowing ample time for citation accumulation over 12 citable years. The year 2020, with seven articles, also demonstrated substantial impact, averaging 130.14 citations per article and 21.69 citations per year, reflecting the high relevance of publications during this period, possibly driven by research on EVs for inflammatory conditions like COVID-19. In contrast, more recent years, such as 2024 (5.50 citations per article) and 2025 (0.57 citations per article), showed lower citation metrics, attributable to their shorter citable periods (2 and 1 year, respectively).

The number of publications grew notably from 2018 onward, with eight articles in 2018, rising to 10 in 2021, 12 in 2022, and peaking at 20 in 2023 and 2024. This surge aligns with advancements in EV isolation techniques and clinical trial initiations. The mean total citations per year peaked in 2014 (49.92), followed by 2018 (21.98) and 2020 (21.69), indicating periods of high scholarly influence. The decline in mean citations per year in later years (e.g., 2.75 in 2024, 0.57 in 2025) reflects the time lag required for citations to accumulate.

**Figure 2:**
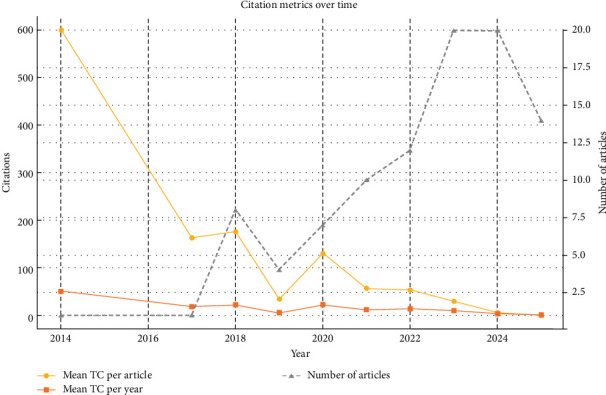
Annual scientific output and citation trends in mesenchymal stem cell-derived extracellular vesicles research. Number of published articles per year (gray line), mean total citations per article (orange line), and mean total citations per year (red line).

Figure 2 illustrates these trends, with the gray line representing the annual number of published articles, the orange line depicting the mean total citations per article, and the red line showing the mean total citations per year. The figure highlights the exponential growth in publication output and the fluctuating citation impact, with early years (2014–2018) showing higher per-article citations due to longer citable periods, while recent years (2023–2025) reflect increased productivity but lower citation metrics due to recency.

### 3.3. Document Content and Keyword Analysis

The dataset comprised 285 author-provided keywords, offering a comprehensive view of research trends and thematic priorities in MSC-derived EV research. Temporal analysis of author keyword frequency showed a marked evolution in research focus from 2017 to 2025. In 2017, “extracellular vesicles” and “exosomes” each appeared once, alongside “mesenchymal stromal cells,” marking the field's early stages. By 2018, frequencies increased, with “extracellular vesicles” (*n* = 4), “exosomes” (*n* = 3), and “immunomodulation” (*n* = 2) gaining traction. The year 2020 saw increased activity, with “exosomes” reaching eight occurrences and “immunomodulation” at 3, likely driven by research on EVs for COVID-19-related inflammation. From 2021 onward, keyword usage surged, with “extracellular vesicles” growing from nine occurrences in 2021–27 in 2025, “exosomes” from 8 to 26, and “mesenchymal stem cells” from 5 to 22. “Osteoarthritis” became prominent later, reaching six occurrences in 2025, signaling a shift toward regenerative applications. The median years of keyword usage (2022 for “extracellular vesicles,” 2023 for “exosomes” and “mesenchymal stem cells,” 2024 for “osteoarthritis”) indicate these terms became central in recent years, with sustained relevance through 2024–2025.

**Figure 3:**
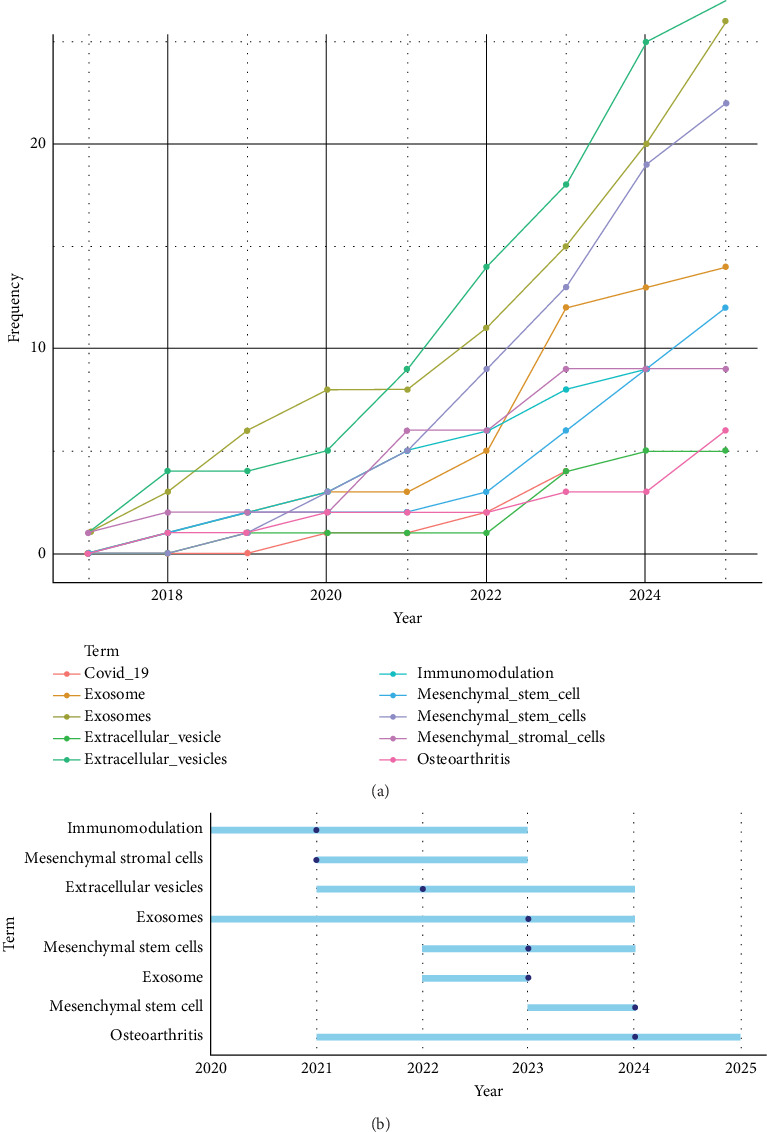
Keyword analysis in mesenchymal stem cell-derived extracellular vesicles research (A) word frequency over time plot of author keywords and (B) trend topics plot of author keywords.

Figure 3A illustrates the word frequency over time for key author keywords, showing the rise of terms like “extracellular vesicles,” “exosomes,” and “mesenchymal stem cells” from 2017 to 2025, with notable increases in “osteoarthritis” and “COVID-19” in later years. Figure 3B depicts trend topics based on author keywords, highlighting the temporal evolution of research themes, from early focus on foundational terms like “exosomes” to recent emphasis on clinical applications such as “osteoarthritis” and “immunomodulation.” These figures underscore the dynamic shift toward translational research, aligning with the increasing number of clinical trials noted in Section 3.8.

### 3.4. Collaboration Patterns

International collaboration was a key driver of MSC-derived EV research, with 22.68% of publications involving cross-border co-authorships, as noted in Section 3.1. The collaboration network, based on co-authorship data, revealed diverse and interconnected partnerships among 17 countries. The United States and China emerged as central nodes, with the strongest bilateral collaboration (frequency = 3), reflecting their leadership in publication output (Section 3.7). The USA also collaborated frequently with Iran (frequency = 2) and Korea (frequency = 2), while China partnered with France (frequency = 2). Other notable collaborations included Iran with Australia, Denmark, Kazakhstan, and the United Kingdom (frequency = 1 each), and India with Germany, Switzerland, Italy, and Turkey (frequency = 1 each). Additional partnerships, such as Brazil–Canada, Korea–Singapore, and Switzerland–Serbia (frequency = 1 each), underscored the global reach of the field.

The collaboration network highlights the United States' pivotal role, with connections to six countries (Chile, Iran, Korea, Netherlands, Serbia, and Switzerland), followed by China and Iran, each with multiple partnerships. These networks facilitate knowledge exchange, resource sharing, and the integration of diverse expertise, particularly in clinical translation and EV characterization. For instance, collaborations between high-income countries (e.g., USA and China) and emerging research hubs (e.g., Iran and India) likely support capacity-building in low- and middle-income countries, addressing global health challenges like inflammatory diseases. The presence of collaborations involving smaller research communities, such as Serbia and Kazakhstan, indicates an inclusive global effort to advance EV therapeutics.

**Figure 4:**
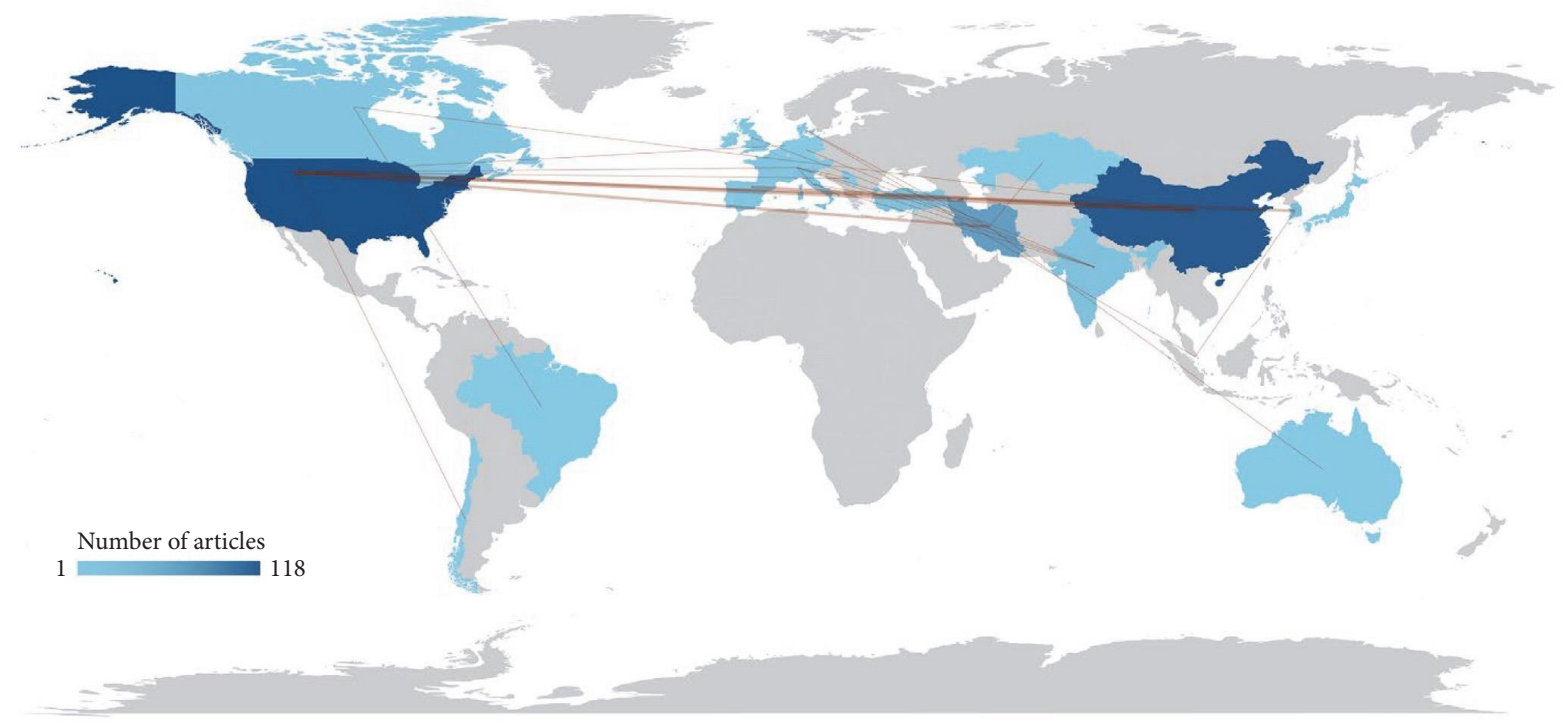
Global collaboration network map of contributing countries in mesenchymal stem cell-derived extracellular vesicles research.

Figure 4 visualizes the global collaboration network map, with nodes representing countries and edges indicating co-authorship frequency. Thicker edges, such as between the USA and China, denote stronger collaborations, while the network's density reflects the field's interconnectedness, supporting the rapid growth observed in publication output (Section 3.2).

### 3.5. Journal Analysis

The bibliometric analysis of MSC-derived EV research identified 70 source journals, as noted in Section 3.1, reflecting the field's interdisciplinary scope. The top 10 journals accounted for a substantial share of the 99 analyzed documents, with Stem Cell Research and Therapy leading with seven publications, followed by Cytotherapy (*n* = 4), and a tie among Frontiers in Immunology, International Journal of Molecular Sciences, Journal of Extracellular Vesicles, and Journal of Nanobiotechnology, each contributing three publications. American Journal of Sports Medicine, Cells, EBioMedicine, and International Journal of Nanomedicine each published two articles, further enriching the publication landscape.

These leading journals, predominantly ranked in the first quartile (Q1) of their respective categories, highlight the high impact and visibility of MSC-derived EV research. Stem Cell Research and Therapy (Q1, Impact Factor ~6.8) serves as a key platform for regenerative medicine, featuring studies on EV applications in tissue repair and immunomodulation. Cytotherapy (Q1, Impact Factor ~5.4) focuses on cellular therapies, publishing research on EV-based clinical advancements. Journal of Extracellular Vesicles (Q1, Impact Factor ~14.0), a specialized high-impact journal, covers EV biogenesis, characterization, and therapeutic potential. Frontiers in Immunology (Q1, Impact Factor ~7.5) and International Journal of Molecular Sciences (Q1, Impact Factor ~5.9) emphasize immunological and molecular aspects, respectively, with publications on EV-mediated immune modulation and molecular mechanisms. Journal of Nanobiotechnology (Q1, Impact Factor ~10.4) underscores the intersection of nanotechnology and EV research, particularly in drug delivery.

The remaining journals, including American Journal of Sports Medicine (Q1, Impact Factor ~6.3), Cells (Q2, Impact Factor ~6.0), EBioMedicine (Q1, Impact Factor ~8.1), and International Journal of Nanomedicine (Q1, Impact Factor ~6.4), address specialized domains such as sports medicine, cellular biology, translational medicine, and nanomedicine. These journals support the field's diverse applications, from osteoarthritis to inflammatory diseases, as identified in Section 3.4. The prevalence of Q1 journals among the top contributors enhances the field's credibility and reach, positioning MSC-derived EV research within high-impact scientific discourse.

**Figure 5:**
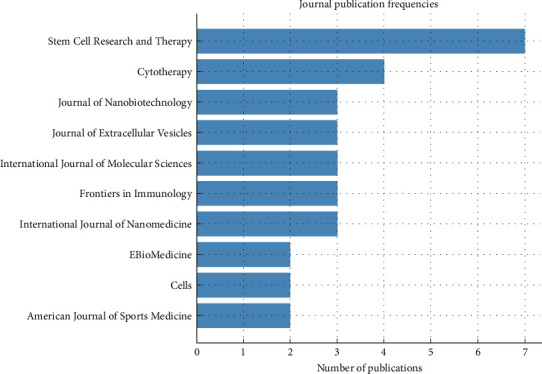
Distribution of publications in top 10 journals for mesenchymal stem cell-derived extracellular vesicles research.

The diversity of publishers, including Springer Nature (Stem Cell Research and Therapy), Elsevier (Cytotherapy, EBioMedicine), Wiley (Journal of Extracellular Vesicles), and MDPI (International Journal of Molecular Sciences, Cells), reflects the field's broad appeal across leading academic platforms. These journals collectively bridge basic science, clinical applications, and technological innovations, aligning with the thematic clusters of cancer therapy, regenerative medicine, and immunotherapy.  Figure 5 illustrates the distribution of publications across the top 10 journals, highlighting Stem Cell Research and Therapy's dominance and the balanced contributions of other high-impact venues, which collectively support the field's growth and clinical translation.

### 3.6. Author Analysis

The bibliometric analysis identified 841 authors contributing to the 99 documents in MSC-derived EV research, as noted in Section 3.1, with an average of 9.92 co-authors per document, reflecting extensive collaborative networks. The top 10 authors, based on publication count, demonstrated significant productivity, with Li Y, Soleimani M, and Wang Y each leading with six articles. Lightner AL, Li M, Liu Y, Wang H, Wang J, Wang L, and Zhang X each contributed four articles, further shaping the field's research landscape.

Fractionalized article counts, which account for co-authorship contributions, provide insight into individual impact. Li Y had the highest fractionalized contribution (0.96), indicating substantial involvement in their six articles, likely as a primary or senior author. Lightner AL followed with 0.72 for four articles, suggesting a significant role in their publications. Soleimani M (0.53 for six articles), Liu Y (0.53 for four articles), and Wang Y (0.49 for six articles) also demonstrated notable contributions, though their lower fractionalized scores reflect broader co-authorship teams. Wang L (0.62 for four articles), Wang H (0.48 for four articles), Zhang X (0.32 for four articles), Wang J (0.31 for four articles), and Li M (0.23 for four articles) had varying levels of fractionalized impact, indicating diverse roles within collaborative efforts.

**Figure 6:**
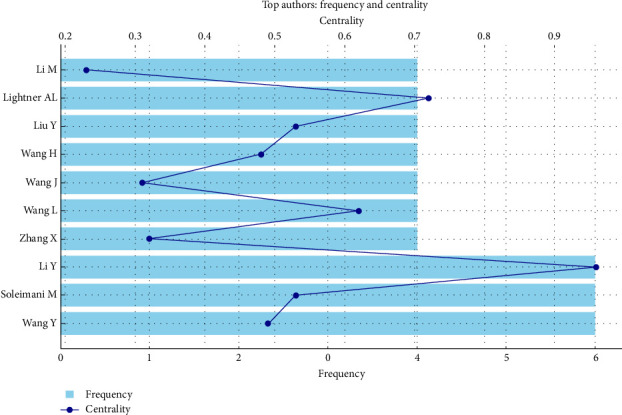
Distribution of publications and fractionalized contributions among top 10 authors in mesenchymal stem cell-derived extracellular vesicles research.

Figure 6 illustrates the distribution of publications among the top 10 authors, highlighting Li Y, Soleimani M, and Wang Y's dominance with six articles each, alongside the balanced contributions of authors with four articles. The figure also visualizes fractionalized contributions, emphasizing Li Y's and Lightner AL's significant individual impact within collaborative frameworks, reinforcing the field's reliance on both teamwork and key individual contributions.

### 3.7. Characteristics of Included Studies

This section summarizes the characteristics of studies included in a bibliometric analysis of MSC-EVs and exosomes as therapeutic agents across human, animal, and in vitro settings. The analysis categorized into human clinical trials (*n* = 29, Table 2), case reports (*n* = 6, Table 3), and animal models (*n* = 50, Table 4) published between 2014 and 2025. Human studies primarily focus on phase I/II clinical trials, case series, and observational studies, evaluating the safety and efficacy of MSC-derived EVs for conditions such as spinal cord injury, osteoarthritis, COVID-19, Alzheimer's disease, and skin disorders. These studies typically employ randomized controlled, open-label, or blinded designs, with interventions including intrathecal, intra-articular, topical, or nebulized administration of EVs from sources like human umbilical cord, adipose, or bone marrow MSCs. Outcomes often highlight safety, reduced inflammation, and improved clinical parameters, though some studies report no significant improvement over placebo.

### 3.8. EV Isolation and Characterization Compliance With International Society for EVs (ISEV) Guidelines

To evaluate methodological rigor and standardization in EV research, all included clinical studies were assessed for their EV isolation techniques, protein marker characterization, and alignment with the ISEV guidelines, namely the 2018 Minimal Information for Studies of EVs (MISEV2018) [41] and the updated 2023 nomenclature guidance [42] (Table 3).

The assessment criteria included: (i) the isolation method employed (e.g., ultracentrifugation, tangential flow filtration, and precipitation), (ii) reporting of at least three positive protein markers from two categories (e.g., tetraspanins such as CD9, CD63, and CD81; cytosolic proteins such as TSG101 and Alix), (iii) inclusion of at least one negative protein marker to confirm purity, and (iv) adherence to the recommended nomenclature and transparent methodological reporting.

Table 3 summarizes these parameters for each study. Of the 27 clinical studies analyzed, only a minority achieved full compliance with MISEV2018 standards, reporting ≥3 positive markers and at least one negative marker alongside particle characterization. The majority demonstrated partial compliance, typically due to omission of negative markers despite reporting multiple positive markers. A subset of studies provided insufficient methodological detail to confirm alignment with ISEV recommendations, particularly regarding marker profiles and particle heterogeneity assessment.

**Figure 7:**
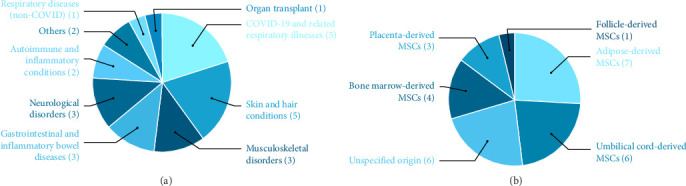
Distribution of mesenchymal stem cell-derived exosome applications for clinical trials and observational studies of human clinical: (A) by different disease categories, (B) by different MSC-EVs sources.

The analysis of clinical studies revealed distinct trends in the application of MSC-EVs across different disease categories (Figure 7A). Respiratory diseases, particularly COVID-19 and related illnesses (*n* = 5), were the most frequently studied, followed by skin and hair conditions (*n* = 5) and autoimmune/inflammatory disorders (*n* = 2). Among MSC sources, adipose tissue (*n* = 7) and bone marrow (*n* = 4) were the most commonly utilized, while placenta (*n* = 3) and umbilical cord (*n* = 6) also demonstrated significant use (Figure 7B). This distribution highlights the broad therapeutic potential of exosomes, with a strong emphasis on inflammatory and regenerative applications.

The outcomes of six case reports investigating the therapeutic potential of MSC-EVs or secretome in diverse clinical conditions are listed in Table 4. The studies demonstrate promising results across multiple pathologies, including interstitial lung disease (ILD), peripheral nerve injury, dental pulp regeneration, ischemic stroke, chronic ulcers, and traumatic brain injury (TBI).

Animal studies utilize models such as ischemic stroke, myocardial infarction, liver fibrosis, and osteoarthritis, predominantly in mice, rats, or pigs, to assess EV-mediated effects on tissue repair, inflammation, and functional recovery (Table 5). These studies frequently demonstrate enhanced regeneration, immunomodulation, and targeted delivery, with mechanisms involving specific miRNAs, proteins, or signaling pathways like NAMPT/SIRT1/FOXO1 or TLR2/IRAK1/NFκB.

## 4. Discussion

The bibliometric analysis of MSC-EVs research reveals a dynamic and rapidly evolving field with significant potential for clinical translation. The findings highlight the increasing interest in MSC-derived EVs as therapeutic agents, driven by their regenerative, immunomodulatory, and targeted delivery capabilities. The analysis demonstrates a robust pipeline of MSC-derived EV research, with 29 clinical trials, six case reports, and 50 animal studies spanning diverse conditions such as COVID-19, osteoarthritis, spinal cord injury, and skin disorders. The prominence of respiratory diseases, particularly COVID-19, in clinical trials underscores the urgency of addressing inflammatory conditions, where EVs have shown promise in reducing mortality and improving oxygenation [25, 32]. Similarly, the focus on regenerative applications, such as osteoarthritis and cartilage repair [12, 29], highlights EVs' potential to address unmet needs in musculoskeletal disorders. The variety of administration routes—intrathecal, intra-articular, topical, and nebulized—demonstrates the versatility of EVs, enabling tailored therapeutic strategies.

The predominance of adipose tissue and bone marrow as MSC sources reflects their established use due to accessibility and well-characterized EV profiles. However, the increasing utilization of umbilical cord and placental MSCs suggests a shift toward scalable, allogeneic sources, which could enhance clinical feasibility by reducing variability and production costs. Animal studies further elucidate mechanisms, such as miRNA-mediated immunomodulation and signaling pathways, providing a foundation for optimizing EV-based therapies. These insights are critical for translating preclinical findings into clinical practice, particularly in designing trials that target specific molecular pathways.

Comparative analyses of MSC-derived EVs from different tissue sources have demonstrated that source origin influences their molecular cargo, immunomodulatory potency, and regenerative capacity. Bone marrow MSC-EVs are often enriched in growth factors and anti-inflammatory cytokines, showing efficacy in musculoskeletal and cardiovascular models [88]. Adipose MSC-EVs tend to yield higher particle concentrations and are rich in angiogenic and matrix-remodeling proteins, supporting wound healing and skin regeneration [89]. Umbilical cord MSC-EVs have shown strong anti-inflammatory and neuroprotective effects, likely due to abundant miRNAs targeting inflammatory pathways, and are widely used for pulmonary and neurological indications [90]. Placenta-derived MSC-EVs exhibit potent immunosuppressive and antifibrotic activities, making them promising for autoimmune and fibrotic disorders [91]. These differences underscore the need to consider MSC tissue origin when designing EV-based therapies, as the choice of source can influence both the mechanistic pathways engaged and the range of clinical applications.

The high degree of international collaboration (22.68% of publications) and the central roles of the United States and China in co-authorship networks indicate a global effort to advance MSC-derived EV research. These collaborations facilitate knowledge exchange, resource sharing, and capacity-building, particularly in emerging research hubs like Iran and India. For instance, partnerships between high-income countries and low- to middle-income countries (e.g., USA–Iran or China–France) support the development of EV therapeutics for global health challenges, such as inflammatory and regenerative diseases. The inclusion of smaller research communities (e.g., Serbia and Kazakhstan) further enhances the field's inclusivity, ensuring diverse perspectives in clinical translation.

These networks are vital for standardizing EV isolation, characterization, and therapeutic protocols, which remain challenges in the field. Collaborative efforts can also address regulatory hurdles, such as harmonizing guidelines for EV-based therapies across jurisdictions. The high-impact journals identified (e.g., Stem Cell Research and Therapy and Journal of Extracellular Vesicles) serve as platforms for disseminating these collaborative outcomes, amplifying the field's visibility and credibility.

The temporal evolution of keywords from foundational terms like “exosomes” to clinical applications like “osteoarthritis” and “immunomodulation” reflects a shift toward translational research. This trend aligns with the surge in publications from 2018 onward, driven by advancements in EV isolation techniques and clinical trial initiations. The high citation impact of early publications (e.g., 2014, 599 citations per article) suggests a strong foundation, while recent studies (2023–2025) indicate ongoing productivity despite lower citations due to recency.

Future research should prioritize addressing gaps identified in the analysis, such as the need for larger, phase III/IV clinical trials to establish efficacy and long-term safety. The current focus on phase I/II trials highlights safety but lacks robust comparative effectiveness data. Additionally, standardizing EV nomenclature (e.g., exosomes vs. EVs) and characterization methods will enhance reproducibility and regulatory approval. The interdisciplinary nature of the field, as evidenced by the diversity of journals, supports integrating nanotechnology, immunotherapy, and regenerative medicine to develop next-generation EV therapies, such as engineered EVs with enhanced targeting or payload delivery.

Our evaluation of included clinical studies against the MISEV2018 guidelines [41] and the 2023 ISEV nomenclature update [42] revealed that only a small proportion of trials achieved full compliance, reporting ≥3 positive protein markers from at least two categories and at least one negative marker, alongside detailed particle characterization. Most studies were partially compliant, typically omitting negative markers or providing limited methodological transparency, while some offered insufficient information to assess adherence. This inconsistency complicates cross-study comparison, bibliometric mapping, and meta-analytic synthesis, and may limit reproducibility and regulatory acceptance of MSC-EV therapies. Greater alignment with ISEV standards—particularly in the accurate use of nomenclature, transparent reporting of isolation and characterization methods, and adoption of negative controls—is essential to improve data comparability and facilitate clinical translation. Future studies should incorporate full MISEV2018-compliant workflows and updated 2023 terminology to enhance scientific rigor and regulatory readiness in EV research.

Analysis of the included studies revealed that MSC-derived EV research has focused on several key disease categories, each supported by distinct mechanistic and translational evidence. Respiratory diseases—particularly COVID-19-related acute respiratory distress syndrome (ARDS)—represent the most frequent clinical application, driven by the urgent need for anti-inflammatory and immunomodulatory interventions. EVs from bone marrow, adipose, and placental MSCs have been shown to reduce pro-inflammatory cytokines and improve oxygenation, findings that are consistent between preclinical ARDS models and early-phase clinical trials [92].

Musculoskeletal disorders, including osteoarthritis and cartilage defects, are the second most frequently targeted category. Preclinical models demonstrate that MSC-EVs promote chondrocyte proliferation, suppress matrix degradation, and enhance cartilage matrix synthesis, mechanisms that are now being validated in phase I/II trials showing improvements in pain and MRI cartilage scores.

Neurological conditions—spinal cord injury, Alzheimer's disease, and ischemic stroke—are emerging as important therapeutic targets. Preclinical studies indicate neuroprotective effects through miRNA-mediated suppression of apoptosis, promotion of axonal regeneration, and modulation of neuroinflammation, though human trials remain in early stages and require long-term safety follow-up.

Dermatological and wound-healing applications leverage the angiogenic and matrix-remodeling cargo of MSC-EVs, with promising results in preclinical skin injury models and small clinical case series. Similarly, autoimmune and inflammatory disorders such as Crohn's-related perianal fistula and Sjögren's syndrome-related dry eye have demonstrated clinical improvements consistent with EV-driven immune modulation.

These patterns highlight both the breadth of MSC-EV research and the areas of greatest translational maturity. COVID-19 and osteoarthritis have advanced furthest along the clinical pathway, while neurological and autoimmune indications remain promising but require larger, controlled trials. Future research should prioritize indications with strong preclinical efficacy and preliminary human safety data, adopt standardized outcome measures to facilitate meta-analysis, and explore engineered EVs with enhanced targeting for disease-specific delivery.

### 4.1. Limitations

The study's reliance on WoS, Scopus, and PubMed may have excluded relevant publications from other databases or gray literature, potentially underrepresenting the field's scope. The exclusion of non-English articles and specific study types (e.g., reviews and pilot studies) may have limited the analysis's comprehensiveness, particularly for emerging research in non-English-speaking regions. The partial year data for 2025 (upto March) likely underestimates recent publication trends, affecting citation metrics and annual growth estimates. Additionally, the bibliometric approach, while effective for mapping trends, does not assess study quality or clinical outcomes, necessitating complementary systematic reviews for deeper insights.

## 5. Conclusion

MSC-derived EV research is a rapidly growing field with significant potential for clinical impact, particularly in inflammatory and regenerative therapies. The global collaboration networks, high-impact publications, and diverse clinical applications underscore the field's maturity and promise. However, challenges such as standardization, larger-scale trials, and comprehensive literature inclusion must be addressed to fully realize EV-based therapeutics' potential. These findings provide a roadmap for researchers, clinicians, and policymakers to advance MSC-derived EV therapies, fostering innovation and global health solutions.

## Figures and Tables

**Table 1:** Query table for bibliometric analysis of mesenchymal stem cell-derived extracellular vesicles research.

Code	Queries
#1	“Extracellular vesicles” or “exosomes” or “extracellular vesicle” or “vesicle, extracellular” or “vesicles, extracellular” or “exovesicles” or “exovesicle”

#2	“Mesenchymal stem cells” or “stem cell, mesenchymal” or “mesenchymal stem cell” or “stem cells, mesenchymal” or “mesenchymal stromal cells” or “mesenchymal stromal cell” or “stromal cell, mesenchymal” or “stromal cells, mesenchymal” or “Wharton jelly cells” or “Wharton's jelly cells” or “Wharton's jelly cell” or “Whartons jelly cells” or “bone marrow stromal cells” or “bone marrow stromal cell” or “bone marrow stromal cells, multipotent” or “multipotent bone marrow stromal cell” or “multipotent bone marrow stromal cells” or “bone marrow stromal stem cells” or “mesenchymal progenitor cell” or “mesenchymal progenitor cells” or “progenitor cell, mesenchymal” or “progenitor cells, mesenchymal” or “multipotent mesenchymal stromal cells” or “mesenchymal stromal cells, multipotent” or “multipotent mesenchymal stromal cell” or “bone marrow mesenchymal stem cells” or “bone marrow mesenchymal stem cell” or “adipose-derived mesenchymal stem cells” or “adipose derived mesenchymal stem cells” or “adipose-derived mesenchymal stromal cells” or “adipose derived mesenchymal stromal cells” or “mesenchymal stem cells, adipose-derived” or “mesenchymal stem cells, adipose derived” or “adipose tissue-derived mesenchymal stromal cell” or “adipose tissue derived mesenchymal stromal cell” or “adipose tissue-derived mesenchymal stromal cells” or “adipose tissue derived mesenchymal stromal cells” or “adipose tissue-derived mesenchymal stem cell” or “adipose tissue derived mesenchymal stem cell” or “adipose tissue-derived mesenchymal stem cells” or “adipose tissue derived mesenchymal stem cells” or “adipose-derived mesenchymal stem cell” or “adipose derived mesenchymal stem cell”

#3	“Clinical trial” or “adaptive clinical trial” or “clinical trial, phase I” or “clinical trial, phase II” or “clinical trial, phase III” or “clinical trial, phase IV” or “controlled clinical trial” or “human challenge trials” or “randomized controlled trial” or “observational study”

#4	#1 And #2 and #3 (filter: english, article)

**Table 2:** A summary of clinical trials and observational studies of human clinical applications of mesenchymal stem cell-derived extracellular vesicles.

Author, reference	Year	Study design	Phase of study	Population	Intervention	Main outcomes
Akhlaghpasand et al. [6]	2024	Single-arm, open-label, and clinical trial	Phase I	Nine patients with complete subacute spinal cord injury	Intrathecal injection of allogeneic human umbilical cord MSC-derived exosomes	Safe; significant improvements in ASIA pinprick (*p*=0.039), light touch (*p*=0.038), SCIM III (*p*=0.027), and NBD scores (*p*=0.042) at 12 months
Bolandnazar et al. [7]	2024	Randomized, triple-blind, and placebo-controlled clinical trial	Not reported	31 Patients with bilateral knee osteoarthritis (grade 2 or 3)	Intra-articular injection of placental MSC-derived EVs vs. saline	Safe; no significant improvement in VAS, WOMAC, lequesne scores, or MRI findings compared to placebo
Chandran et al. [8]	2025	Open-label study	Phase I	10 Healthy adult volunteers with psoriasis	Topical MSC exosome ointment (PTD2021P) thrice daily for 20 days	Well-tolerated; no serious adverse events; no abnormalities in laboratory parameters or visual skin assessments
Chu et al. [9]	2022	Pilot trial	Not applicable	Seven patients with COVID-19 pneumonia	Nebulization of umbilical cord MSC-derived exosomes	Safe; promoted pulmonary lesion absorption and reduced hospitalization duration in mild cases
Crose et al. [10]	2024	Pilot safety study	Not applicable	10 ALS patients	Two 10-mL intravenous infusions of bone marrow MSC-derived EVs 1 month apart	Safe; no serious adverse events; 30% showed no decline in ALSFRS-R scores
Estupinan et al. [11]	2025	Investigator-blinded, split-face, and noninferiority trial	Not reported	Patients with mild to moderate photoaged facial skin	Topical adipose MSC-derived exosomes vs. platelet-rich plasma with radiofrequency microneedling	Equal improvement in wrinkling, dyschromia, erythema, texture; increased collagen I and glycosaminoglycans
Figueroa-Valdes et al. [12]	2025	First-in-human and open-label study	Phase I	Patients with knee osteoarthritis	Intra-articular umbilical cord MSC-derived sEVs	Safe; no adverse effects after 12 months; promoted cartilage regeneration and polarized macrophages to M2b-like phenotype
Gentile et al. [13]	2025	Multicentric, observational, and evaluator-blinded study	Not applicable	60 AGA patients (40 MPHL, 20 FPHL)	Micrografts with follicle MSC-derived EVs	Increased hair density (28 ± 4 hairs/cm^2^ FPHL, 30 ± 5 hairs/cm^2^ MPHL); safe, viable for hair loss treatment
Habibi et al. [14]	2025	Triple-blinded and randomized trial	Phase I/II	16 Eyes with Sjogren's syndrome-related dry eye	Topical MSC-derived exosomes vs. PBS	Improved OSDI, tear secretion, fluorescein scores, and tear-film break-up time; reduced IL-6 and MMP-9
Hadizadeh et al. [15]	2024	Nonrandomized and nonblinded clinical trial	Phase II	20 Patients with refractory Crohn's perianal fistula	Three injections of MSC-derived exosomes into fistula tracts	60% achieved complete fistula closure; reduced inflammation and enhanced tissue regeneration
Harrell et al. [16]	2020	Clinical trial	Not reported	COPD patients	MSC-derived product “exo-d-MAPPS”	Improved respiratory function, reduced inflammatory cytokines, expanded immunosuppressive cells, and well tolerated in patients
Jiang et al. [17]	2025	Double-blind, randomized and self-controlled clinical trial	Not reported	43 Traumatic patients with impacted third molar extraction	Lyophilized MSC-derived apoptotic vesicles (MSC-apoVs)	Safe and effective for hemostasis and bone regeneration; improved alveolar bone regeneration
Li et al. [18]	2025	Randomized, single-blind, and placebo-controlled clinical trial	Phase I/II	24 Pulmonary fibrosis patients	Nebulized hUCMSC-derived extracellular vesicles (hUCMSC-EVs)	Improved lung function indices, respiratory health status, and CT scans; well tolerated
Lightner et al. [19]	2023	Randomized and placebo-controlled dosing clinical trial	Phase II	102 COVID-19 patients with moderate to severe ARDS	Bone marrow MSC-derived extracellular vesicles (ExoFlo)	Reduced mortality, improved ventilation-free days, and no adverse events
Lightner et al. [20]	2024	Case series (emergency IND use)	Not applicable	Seven solid abdominal organ transplant patients	MSC-derived extracellular vesicles (EVs)	Improved graft inflammation/rejection and no adverse events
Nazari et al. [21]	2022	Clinical trial	Phase I	Five IBD patients with refractory perianal fistulas	MSC-derived exosomes	Safe and effective; complete healing in three patients.
O'Brien et al. [22]	2021	Preclinical study (in vitro and in vivo)	Not applicable	Patient-specific iCMs and SENECA trial patients	Mitochondria-rich extracellular vesicles (L-EVs)	Improved cardiomyocyte viability and reduced DOX injury
Pak et al. [23]	2023	Clinical trial	Phase I	11 Non-Crohn's patients with complex perianal fistulas	Placenta MSC-derived exosomes	Safe and effective; complete healing in five patients
Park et al. [24]	2023	Randomized and split-face study	Not applicable	28 Individuals with facial skin aging	Adipose tissue stem cell-derived exosome-containing solution (HACS) + microneedling	Improved skin wrinkles, elasticity, hydration, and pigmentation
Sengupta et al. [25]	2020	Prospective nonrandomized open-label cohort study	Not applicable	24 Patients with severe COVID-19 and ARDS	Single 15 mL intravenous dose of bone marrow MSC-derived exosomes (ExoFlo)	Safe; 83% survival; improved oxygenation (PaO_2_/FiO_2_ + 192%) and reduced inflammatory markers
Shi et al. [26]	2021	Preclinical-to-early-clinical safety study	Phase I	24 Healthy volunteers	Nebulized adipose MSC-derived EVs (2–16 × 10^8^ particles)	Safe; improved survival (80%) in *P. aeruginosa*-induced lung injury model; reduced lung inflammation
Svolacchia et al. [27]	2024	Retrospective clinical evaluation study	Not applicable	Patients with dermal wrinkles and facial tissue furrows	Ultrafiltered adipose MSC-derived signaling nanovesicles	Excellent results in skin regeneration; safe and reduced inflammation without cellular debris
Wan et al. [28]	2025	Case series	Not applicable	20 Adults with moderate-to-severe facial atopic dermatitis	Topical adipose MSC-derived exosomes twice daily for 6 weeks	85% achieved ≥1-point vIGA-AD reduction; 58% improved hydration, 42% reduced TEWL, and 70% reduced pruritus
Wang et al. [29]	2025	Randomized, double-blind, and ascending dose clinical trial	Phase I	Patients with osteoarthritis	Intra-articular human umbilical cord MSC-derived exosomes	Safe; reduced inflammation, promoted cartilage regeneration; improved clinical scores, and MRI findings
Xie et al. [30]	2023	Clinical trial	Phase I/II	Patients with mild to moderate Alzheimer's disease	Intranasal adipose MSC-derived exosomes	Safe; improved cognitive function (ADAS-cog −2.33 and MoCA + 2.38); less hippocampal volume loss
Ye et al. [31]	2022	Clinical trial	Not reported	22 Females with sensitive skin	Topical human MSC exosomes	Improved roughness, erythema; normalized TEWL, hydration, sebum, and pH
Zamanian et al. [32]	2024	Double-blind and randomized controlled trial	Not reported	45 Patients with COVID-19 ARDS	Placental MSC-derived sEVs vs. placebo	Reduced mortality (19.04% vs. 54.16%, *p*=0.015); increased time to death (28.06 vs. 11.10 days, *p* < 0.001)
Zarrabi et al. [33]	2023	Randomized controlled trial	Phase II	43 Patients with COVID-19 ARDS	MSCs or MSCs + EVs vs. control	Safe; reduced inflammatory cytokines (IL-6, TNF-α, IFN-γ, and CRP); no mortality in MSC + EV group
Zhu et al. [34]	2022	Single-arm and open-label trial	Phase II	Seven patients with severe COVID-19	Nebulized adipose MSC-derived exosomes (2.0 × 10^9^ vesicles)	Safe; improved lymphocyte counts and pulmonary lesion resolution

**Table 3:** Mesenchymal stem cell-derived extracellular vesicles isolation methods, marker profiles, and alignment of included clinical studies with MISEV 2018 and 2023 nomenclature guidelines.

Author, reference	Isolation procedure	Positive and negative protein markers	Alignment with MISEV 2018 and 2023 nomenclature
Akhlaghpasand et al., [6]	Ultracentrifugation (sequential at 300 × *g*, 2000 × *g*, 10,000 × *g*, and 100,000 × *g*) and filtration through 0.22 µm filter	Positive: CD9 and CD81 negative: not specified	Partial
Bolandnazar et al., [7]	Sequential centrifugation (300 × *g*, 2000× *g*, 10,000 × *g*, and 110,000 × *g*) and elution with PBS	Positive: CD9, CD63, and CD81 negative: not specified	Partial
Chandran et al., [8]	Not detailed in provided text; previously confirmed as endosome-derived exosomes with lipid membranes and proteins	Positive: CD81, CD9, and alix negative: not specified	Partial
Chu et al., [9]	Multiple ultrafiltration steps (centrifugation at 3000 × *g*, 1500 × *g*, 100,000 × *g*, and filtration through 0.22 µm filter)	Positive: CD81, CD9, and flotillin 2 negative: β-actin	Full
Crose et al., [10]	Not detailed in provided text; described as extensively characterized with advanced particle analysis and proteomic evaluation	Positive: not specified negative: not specified	Insufficient
Estupinan et al., [11]	Two-step filtration (ExoSCRT technology) to separate 0.1%−0.5% pure exosomes	Positive: not specified negative: not specified	Insufficient
Gentile et al., [13]	Mechanical disaggregation (rigeneracons device), centrifugation at 1200 rpm, filtration through 0.22 µm filter, and ultrafiltration	Positive: CD9, CD63, CD81, CD44, and CD105 negative: not specified	Partial
Habibi et al., [14]	Ultracentrifugation (sequential at 400 × *g*, 2500 × *g*, and 110,000 × *g*) and filtration through 0.22 µm filter	Positive: CD9, CD63, and CD81 negative: not specified	Partial
Hadizadeh et al., [15]	Ultracentrifugation (100,000 × *g* for 60 min, twice) and filtration	Positive: CD9, CD63, and CD81 negative: not specified	Partial
Harrell et al., [16]	Ultracentrifugation (100,000 × *g* for 70 min), positive selection using µMACS separator with exosome isolation kit (CD9, CD63, and CD81 microbeads)	Positive: CD9, CD63, and CD81 negative: not specified	Partial
Li et al., [18]	Ultracentrifugation (100,000 × *g*), filtration, meeting cGMP and MISEV2018 standards	Positive: CD9, CD63, and CD81 negative: calnexin	Full
Lightner et al., [19]	Not detailed in provided text; meets cGMP standards, characterized for size, quantity, and tetraspanin profile (CD9, CD63, and CD81)	Positive: CD9, CD63, and CD81 negative: not specified	Partial
Nazari et al., [21]	Ultracentrifugation (sequential at 400 × *g* for 10 min, 2500 × *g* for 30 min, and 100,000 × *g* for 120 min twice) and dissolved in PBS	Positive: CD9, CD63, and CD81 negative: not specified	Partial
Pak et al., [23]	Ultracentrifugation (sequential at 400 × *g* for 10 min, 2500 × *g* for 30 min, and 100,000 × *g* for 120 min twice) and dissolved in PBS	Positive: CD9 and CD81 negative: not specified	Partial
Park et al., [24]	Not detailed in provided text; HACS prepared from ASCE + derma signal skin rejuvenation lyophilized vial	Positive: not specified negative: not specified	Insufficient
Sengupta et al., [25]	Not detailed in provided text; processed in FDA-registered facilities meeting cGMP and CGTP standards	Positive: not specified negative: not specified	Insufficient
Shi et al., [26]	Differential centrifugation, 12% PEG incubation, centrifugation at 3000 × *g* for 1 h, ultracentrifugation at 120,000 × *g* for 70 min, and resuspended in saline	Positive: CD9, CD81, CD63, and TSG101 negative: CANX	Full
Svolacchia et al., [27]	Disaggregation (Tonnard method), microfiltration at 20/40 µm, ultrafiltration at 0.20 µm, and stabilized with Skin-B (hyaluronic acid and amino acids)	Positive: CD81 and CD146 negative: not specified	Partial
Wang et al., [29]	Not detailed in provided text; meets MISEV2018 standards and confirmed by TEM and NTA	Positive: CD9, CD63, and TSG101 negative: CALN	Full
Xie et al., [30]	Precipitation with polyethylene glycol, ultracentrifugation, and optimized for cGMP compliance	Positive: CD63, CD81, CD9, and TSG101 negative: CANX	Full
Ye et al., [31]	Ultracentrifugation (sequential at 300 × *g*, 2000 × *g*, 10,000 × *g*, and 100,000 × *g*), filtration through 0.22 µm filter	Positive: CD63, CD9, and TSG101 negative: not specified	Partial
Zamanian et al., [32]	Centrifugation (700 × *g* for 10 min and 9000 × *g* for 30 min), PEG 6000 (8%) incubation for 16 h, centrifugation at 5000 × *g* for 1 h, and ultracentrifugation at 100,000 × *g* for 2 h	Positive: CD81, CD9, and CD63	Partial
Zarrabi et al., [33]	Tangential flow filtration, centrifugation at 3000 × *g* and 20,000 × *g*, filtration through 0.2 µm, and resuspended in saline	Positive: not specified negative: not specified	Insufficient
Zhu et al., [34]	Not detailed in provided text; clinical-grade exosomes characterized for size (~100 nm)	Positive: not specified negative: not specified	Insufficient

**Table 4:** Summary of case reports on mesenchymal stem cell-derived extracellular vesicle therapies.

Author, reference	Year	Patient/condition	Disease/organ system	Intervention	Main outcomes
Assar et al. [35]	2023	55-Year-old woman with systemic sclerosis-related ILD	Respiratory (interstitial lung disease)	Eight doses of placental MSC-EVs	Improved dyspnea, cough, reduced fibrosis on CT, better exercise tolerance, and reduced oxygen need
Civelek et al. [36]	2024	24-Year-old male with total radial nerve injury	Neurological (peripheral nerve)	1 mL MSC-derived exosomes (5 billion microvesicles) applied to nerve graft sites	Excellent motor (M5) and good sensory (S3+) recovery; EMG-confirmed reinnervation
Jafari et al. [37]	2025	40-Year-old man with irreversible pulpitis	Dental/oral	hUCMSC-derived exosomes + chitosan in root canal	No infection; radiographic signs of healing; maintained tooth vitality
Lin et al. [38]	2023	45-Year-old female with acute ischemic stroke	Neurological (central nervous system)	Weekly IV amniotic MSC-secretome for 4 weeks	Increased regulatory T cells; improved paralysis
Messa et al. [39]	2022	Patient with recurrent ischial ulcer	Dermatological/wound healing	Six subcutaneous ExoFlo exosome injections over 8 weeks	Complete ulcer healing
Nabity and Ransom [40]	2025	Patient with severe traumatic brain injury	Neurological (central nervous system)	IV hBM-MSC EVs 3 x/week for 6 months	48%–55% improvement in FIM/FAM scores; enhanced mobility and cognition

**Table 5:** Mechanisms of action and regenerative outcomes of mesenchymal stem cell-derived exosomes in animal models.

Author, reference	Year	Model	Intervention	Main outcomes
Azizsoltani et al. [43]	2023	CCL4-induced liver fibrosis mouse model	Wharton's jelly MSC-derived exosomes loaded with obeticholic acid	Reduced fibrotic indicators, AST/ALT; inactivated hepatic stellate cells; improved ECM remodeling
Barzegar et al. [44]	2021	Transient MCAO mouse model	Intraperitoneal or intravenous placental MSC-derived EVs	Reduced ischemic injury, restored cerebral blood flow; lipid-dependent protection
Bellio et al. [45]	2022	Murine myocardial infarction model	Systemic Wharton's jelly MSC-derived EVs	Improved cardiac function (ejection fraction, contractility, and lusitropy); scalable EV production
Blanco et al. [46]	2023	Cecal ligation and puncture sepsis mouse model	EVs from bone marrow, adipose, or lung MSCs	Reduced lung/kidney damage; bone marrow EVs most effective due to anti-inflammatory proteome
Cai et al. [47]	2023	Chronic rotator cuff tear rat model	Kartogenin-preconditioned bone marrow MSC exosomes	Improved cartilage formation and collagen maturation; enhanced biomechanical properties
Chen et al. [48]	2025	Hind limb ischemia mouse model	Bone marrow MSC-derived apoptotic vesicles	Improved angiogenesis, proliferation, and migration via NAMPT/SIRT1/FOXO1 axis
Chen et al. [49]	2019	Hippocampal damage mouse model	MSC EVs with EP(4) antagonist	Repaired cognition/learning deficiencies; reduced inflammation and microglial infiltration
Deng et al. [50]	2025	Rhabdomyolysis-induced AKI mouse model	Klotho-loaded MSC-derived sEVs	Accelerated renal recovery and reduced injury markers; activated mTOR and MEK1/2 pathways
Eirin et al. [51]	2019	Porcine metabolic syndrome model	MSC-derived EVs from Lean vs. MetS pigs	MetS-EVs increased renal inflammation; Lean-EVs enhanced reparative pathways
Figueroa-Valdes et al. [12]	2025	Mouse OA model	UC-MSC-sEVs	Promoted cartilage regeneration and reduced inflammation
Gonzalez et al. [52]	2023	*E. coli* pneumonia rat model	Nebulized bone marrow MSC-derived conditioned medium	Improved lung function, reduced bacterial load, and inflammatory cytokines
Hai et al. [53]	2018	NOD mouse model of Sjogren's syndrome	iPSC-MSC EVs	Reduced lymphocyte infiltration and serum autoantibody levels
Harman et al. [54]	2018	Equine wound healing model	MSC-secreted PAI-1 and tenascin-C	Improved wound healing and fibroblast migration
Harrell et al. [16]	2020	COPD mouse model	Exo-d-MAPPS (MSC-derived exosomes)	Improved respiratory function and reduced inflammation
Hede et al. [55]	2021	Minipig chondral defect model	MSC-EVs + bone marrow stimulation	Enhanced subchondral bone healing but impaired cartilage repair
Homma et al. [56]	2023	Sheep sepsis model	MSC-EVs	Limited improvement in pulmonary gas exchange
Hu et al. [57]	2024	Cynomolgus monkeys	hUC-MSC exosomes	No adverse effects and safe for clinical use
Jeong et al. [58]	2021	Alzheimer's mouse model	NEP-enhanced hUC-MSC EVs	Improved neurogenesis and anti-inflammation
Lentilhas-Graca et al. [59]	2024	Mouse spinal cord injury model	M(IL-10 + TGF-β1) macrophage secretome	Improved functional recovery and reduced inflammation
Levy et al. [60]	2023	Diabetic wound mouse model	iPSC-derived EVs	Enhanced inflammation resolution and wound healing
Levy et al. [61]	2023	In vitro storage study	MSC-EVs stored at various conditions	Retained bioactivity for 4–6 weeks at −20°C/−80°C
Li et al. [62]	2020	Rabbit osteonecrosis model	BMMSC exosomes	Upregulated PAI-1 and disrupted coagulation in femoral head
Li et al. [18]	2025	Pulmonary fibrosis patients	Nebulized hUC-MSC-EVs	Improved lung function and respiratory health
Liu et al. [63]	2024	Mouse OA model	Infrapatellar fat pad MSC-EVs	Delayed OA progression, reduced pain and inflammation
Liu et al. [64]	2024	Rat BMSC and ASC EVs	EVs from BMSCs and ASCs	Varied angiogenic and immunomodulatory potentials
Lu et al. [65]	2019	Rhesus macaque wound model	Autologous and allogeneic iPSC exosomes	Accelerated wound healing, reduced inflammation
Ma et al. [66]	2024	Colorectal cancer model	BMSC-EVs with MAGI2-AS3	Inhibited cancer progression via MYC protein modulation
Mocchi et al. [67]	2021	Equine musculoskeletal disease	Lyosecretome (freeze-dried MSC secretome)	Validated production process for clinical use
Monguio-Tortajada et al. [68]	2022	Porcine myocardial infarction	cATMSC-EVs in pericardial scaffold	Improved cardiac function and reduced inflammation
Park et al. [69]	2023	Human facial skin aging	ASCE-containing solution + microneedling	Improved skin elasticity, hydration, and pigmentation
Peng et al. [70]	2024	Replicative senescent BMMSCs	Young sEVs from deciduous teeth stem cells	Rejuvenated senescent cells via Drp1-mediated mitochondrial dynamics
Pisano and Besner [71]	2019	Murine NEC model	Stem cell-derived exosomes	Reduced NEC incidence and severity
Shi et al. [26]	2021	*P. aeruginosa*-induced lung injury mouse model	Nebulized adipose MSC-derived EVs	Improved survival (80%), reduced lung inflammation, and histological severity
Shi et al. [72]	2022	MCD-induced NASH mouse model	Human umbilical cord MSC-derived exosomes	Reduced body weight loss and liver damage; promoted anti-inflammatory macrophage phenotype
Sun et al. [73]	2025	5xFAD Alzheimer's disease mouse model	Intranasal BACE1 siRNA and berberine-loaded MSC exosomes	Improved cognitive function, reduced BACE1, Aβ deposition, and inflammation
Sun et al. [74]	2018	Type 2 diabetes mellitus rat model	Human umbilical cord MSC-derived exosomes	Reduced blood glucose, reversed insulin resistance, and restored β-cell function
Taglauer et al. [75]	2021	Heme oxygenase-1 null preeclampsia mouse model	Umbilical cord MSC-derived EVs (MEx)	Improved fetal loss, growth restriction, and placental function; modulated uterine immune environment
Wan et al. [76]	2023	Osteoarthritis mouse model	Photocrosslinking hydrogel-encapsulated MSC exosomes with LRRK2-IN-1	Suppressed catabolism and promoted anabolism; improved cartilage repair
Wang et al. [77]	2024	Streptozotocin-induced diabetic nephropathy mouse model	Exendin-4-enriched umbilical cord MSC exosomes	Improved blood glucose, proteinuria, and kidney damage; increased CD4 + treg cells via Prevotella
Wang et al. [29]	2025	Human knee OA patients	hUC-MSC exosomes	Improved cartilage regeneration and no adverse effects
Webb et al. [78]	2018	Thromboembolic stroke mouse model	Neural stem cell-derived EVs	Improved tissue and functional outcomes; altered systemic immune response
Wu et al. [79]	2025	Osteoarthritis mouse model	miR-7704-modified umbilical cord MSC EVs	Improved walking capacity and cartilage morphology; reduced MMP13 expression
Xiao et al. [80]	2021	Natural aging and type-2 diabetes mouse wound-healing models	MSC-derived sEVs	Mitigated endothelial senescence and stimulated angiogenesis via miR-146 a/Src
Yang et al. [81]	2020	Atopic dermatitis mouse model	SOD3-transduced MSC EVs	Reduced symptoms and immune cell activation; EVs carried SOD3 protein
Yudintceva et al. [82]	2024	Rabbit renal tuberculosis model	MSC-derived EVs with anti-tuberculosis treatment	Reduced inflammation and kidney damage; increased anti-inflammatory cytokines
Zhang et al. [83]	2022	EAE and cuprizone demyelination mouse models	Bone marrow MSC-derived exosomes	Promoted remyelination and reduced neuroinflammation via TLR2/IRAK1/NFκB inhibition
Zhang et al. [84]	2020	Myocardial ischemia/reperfusion injury mouse model	Monocyte mimic-bioinspired MSC-derived EVs	Enhanced myocardial targeting, improved endothelial maturation, and macrophage modulation
Zhang et al. [85]	2022	Osteochondral defect micropig model	Human MSC exosomes with hyaluronic acid	Improved cartilage and subchondral bone repair; enhanced biomechanical properties
Zhou et al. [86]	2022	GVHD-associated dry eye mouse model	MSC-derived exosomes	Alleviated dry eye; reprogramed M1–M2 macrophages via miR-204
Zhou et al. [87]	2023	Ischemic stroke mouse model	BDNF-loaded MSC-derived sEVs	Enhanced neural repair and reduced infarct volume; upregulated neuroprotection genes

## Data Availability

The data that support the findings of this study are available from the corresponding author upon reasonable request.
